# Helper T cells and chemokines in elderly asthma—Mechanisms of airway inflammation and remodeling: A review

**DOI:** 10.17305/bb.2026.13875

**Published:** 2026-03-10

**Authors:** Youhua Wu

**Affiliations:** 1 Department of Geriatrics, Dalian Third People’s Hospital Affiliated to Dalian University of Technology, Dalian, Liaoning, China.

**Keywords:** Elderly asthma, helper T cells, chemokines, mechanism of action, airway inflammation, correlation

## Abstract

Elderly bronchial asthma is a heterogeneous, often non-atopic disorder characterized by airway inflammation and remodeling influenced by age-related immune dysregulation. This review aims to elucidate the roles of helper T cells (Th cells) and chemokine networks in driving elderly asthma and to emphasize implications for precise diagnosis and targeted therapy. We performed a narrative synthesis of studies from PubMed and Web of Science (January 2020–December 2025) utilizing the keywords “elderly asthma,” “helper T cells,” “chemokines,” “airway inflammation,” and “immunosenescence,” focusing on human cohorts aged ≥65 years and aged animal models. The literature reveals that the pathogenesis of elderly asthma is characterized by an imbalance between T helper 1 (Th1) and T helper 2 (Th2) responses, enhanced T helper 17 (Th17) activity, and diminished regulatory T cell (Treg) function, changes that are exacerbated by immunosenescence. Key chemokine axes—including C-C motif chemokine ligand 11 (CCL11)/C-C chemokine receptor 3 (CCR3), C-X-C motif chemokine ligand 8 (CXCL8)/C-X-C chemokine receptor 1 (CXCR1) and C-X-C chemokine receptor 2 (CXCR2), C-C motif chemokine ligand 2 (CCL2)/C-C chemokine receptor 2 (CCR2), C-X3-C motif chemokine ligand 1 (CX3CL1)/C-X3-C chemokine receptor 1 (CX3CR1), and stromal cell-derived factor 1 (SDF-1)/C-X-C motif chemokine ligand 12 (CXCL12)/C-X-C chemokine receptor 4 (CXCR4)—facilitate the recruitment of eosinophils, neutrophils, and monocytes/macrophages, thereby sustaining airway inflammation and remodeling. Overall, Th-chemokine circuits represent actionable targets for biomarker-guided, personalized treatment; however, further mechanistic and clinical validation studies specific to the elderly population are crucial for effective translation into practice.

## Introduction

Elderly asthma is a heterogeneous disease characterized by airway obstruction, increased airway responsiveness, airway remodeling, and inflammatory processes. Its complex pathogenesis can lead to severe complications, such as respiratory failure and pulmonary encephalopathy, which pose significant risks to patients’ lives [1, 2]. Thus, early diagnosis of elderly asthma is essential for enhancing clinical efficacy and prognosis. In this review, “elderly” refers to individuals aged 65 years or older, in line with the World Health Organization’s definition and the inclusion criteria of most epidemiological studies on late-onset asthma. The disease arises from the interaction of various factors, including eosinophils (EOS), mast cells (MCs), and neutrophils, with airway inflammation serving as a central mechanism in its pathogenesis. T helper (Th) cells are involved at all stages of the disease process in elderly asthma. Chemokines facilitate the migration, activation, and degranulation of inflammatory cells, with C-C motif chemokines (CC chemokines) and their receptors playing crucial roles in airway inflammation’s development and emerging as important therapeutic targets for elderly asthma [3, 4]. Recent extensive research [5, 6] has underscored the significance of helper T (Th) cells and related chemokines in the pathogenesis of elderly asthma. This article reviews the current literature on the roles of Th cells and chemokines in the disease’s progression.

For this narrative review, we conducted a search of PubMed and Web of Science for articles published between January 2020 and December 2025, using keywords such as “elderly asthma,” “helper T cells,” “chemokines,” “airway inflammation,” and “immunosenescence.” We prioritized original studies and high-quality reviews focusing on human cohorts aged ≥65 years or aged animal models; landmark older papers were included only when they provided foundational mechanistic concepts not superseded by recent work.

## Role of Th cells in the pathogenesis of elderly asthma

Elderly asthma fundamentally differs from childhood- or adult-onset asthma in several clinically and pathophysiologically relevant dimensions. Age of onset is a critical differentiator; late-onset asthma (first presentation after age 65) is more prevalent in the elderly and is often non-atopic, characterized by normal or low serum immunoglobulin E (IgE) and negative allergen skin tests [7–9]. This contrasts sharply with the strong T helper 2 (Th2) cells-driven allergic phenotype typical of early-onset disease [10]. Additionally, the inflammatory phenotype in elderly asthma is frequently mixed or neutrophilic rather than purely eosinophilic [11, 12], and severe cases are commonly associated with glucocorticoid resistance [13], a feature less prevalent in younger populations [14]. Elderly patients also often present with multiple comorbidities (e.g., chronic rhinosinusitis, gastroesophageal reflux, cardiovascular disease, and osteoporosis), complicating diagnosis, treatment, and disease monitoring [15, 16]. Furthermore, airflow limitation in the elderly may reflect not only asthma-related remodeling but also accelerated lung aging, including loss of elastic recoil and age-related decline in respiratory muscle strength [17, 18], making it challenging to distinguish from chronic obstructive pulmonary disease [19].

At the immunological level, the classical Th2-centric paradigm is insufficient to explain the complexity of elderly asthma [20]. Immunosenescence—the age-associated remodeling of the immune system—creates a permissive background characterized by a contracted naïve T-cell repertoire, expanded memory T-cell pools, accumulation of terminally differentiated senescent T cells, and a chronic low-grade inflammatory state termed “inflammaging” [21, 22]. These alterations shift the balance from pure T helper 2 (Th2) responses to mixed T helper 1 (Th1)/T helper 17 (Th17) profiles, thereby enhancing neutrophilic inflammation and impairing regulatory T-cell (Treg) function [23, 24]. Consequently, elderly asthma is better conceptualized as a senescence-associated inflammatory disorder rather than a purely allergic disease [25]. This reframing carries significant therapeutic implications. Agents that are highly effective in treating atopic, eosinophilic childhood asthma, such as anti-IL-5/interleukin-5 receptor alpha (IL-5Rα) biologics, may produce suboptimal responses in elderly patients who do not exhibit a robust type 2 immune signature [26]. In contrast, targeting neutrophil-recruiting chemokines or senescence-associated pathways may provide greater therapeutic benefits in this demographic [27].

Elderly asthma represents a distinct pathobiological entity that cannot be adequately understood through extrapolation from younger cohorts. The following sections examine the specific roles of Th cell subsets and chemokine networks within this unique geriatric framework.

### Relationship between Th cells and the pathogenesis of elderly asthma

Th cells are increasingly recognized for their central role in the pathogenesis of elderly asthma. Their functional imbalance underlies the immunological basis for the heterogeneity of airway inflammation and the variability in treatment responses observed in elderly asthmatic patients. While the Th1/Th2 imbalance is a classic paradigm in asthma pathogenesis, this model presents a more complex picture in the elderly population. Recent studies confirm that although Th2 cells and the interleukin (IL)-4, IL-5, and IL-13 cytokine cascade they drive remain active in some elderly patients, therapies solely inhibiting the Th2 pathway often yield suboptimal results, indicating the involvement of other key pathways [7]. To summarize the current evidence regarding the roles of Th cell subsets and chemokines in elderly asthma, Table 1 provides an overview of key Th cell subsets, their cytokine profiles, associated chemokines, and their contributions to airway inflammation and remodeling.

**Table 1:** Summary of evidence on Th cell subsets and chemokines in elderly asthma

**Th cell subset**	**Key cytokines**	**Associated chemokines**	**Role in elderly asthma**
Th1	IFN-γ, IL-2, TNF-α	CCL2, CXCL9, CXCL10	Promotes pro-inflammatory responses, associated with neutrophilic inflammation. Often implicated in severe, steroid-resistant asthma in elderly patients.
Th2	IL-4, IL-5, IL-13	CCL11, CCL17, CCL22	Drives eosinophilic inflammation, mucus production, and airway remodeling. A prominent feature in mild to moderate asthma in the elderly.
Th17	IL-17A, IL-22	CXCL8 (IL-8), CCL20	Enhances neutrophilic inflammation and airway remodeling. Linked to severe asthma and glucocorticoid resistance, particularly in older adults.
Treg	TGF-β, IL-10	CCL18, CX3CL1	Regulates immune tolerance and suppresses excessive inflammation. Decline in function is associated with increased asthma severity in the elderly.

Abbreviations: Th1: T helper 1 cells; Th2: T helper 2 cells; Th17: T helper 17 cells; Treg: Regulatory T cells; IFN-γ: Interferon gamma; TNF-α: Tumor necrosis factor alpha; TGF-β: Transforming growth factor beta; CCL2: C-C motif chemokine ligand 2; CXCL9: C-X-C motif chemokine ligand 9; CXCL10: C-X-C motif chemokine ligand 10; CCL11: C-C motif chemokine ligand 11; CCL17: C-C motif chemokine ligand 17; CCL22: C-C motif chemokine ligand 22; CXCL8: C-X-C motif chemokine ligand 8; CCL20: C-C motif chemokine ligand 20; CCL18: C-C motif chemokine ligand 18; CX3CL1: C-X3-C motif chemokine ligand 1.

Among these key pathways, Th17 cell-mediated neutrophilic inflammation has been shown to play a prominent role in elderly asthma, particularly in severe and steroid-resistant forms. This perspective is supported by international research. Smith et al. [8], using polychromatic flow cytometry on peripheral blood from a cohort of asthmatic patients aged ≥65 years, demonstrated that CD4+ T cells from elderly asthmatic patients exhibit a greater propensity to differentiate into the Th17 lineage. Their study further revealed that this differentiation is closely associated with alterations in the respiratory microbiome, elucidating how aging factors reshape the microenvironment of T-cell immune responses.

In addition to Th2 and Th17 cells, defective Treg cell function critically contributes to the breakdown of immune homeostasis in elderly asthma. As central players in maintaining immune tolerance, the age-related decline in both the number and function of Treg cells impairs their inhibitory control over effector T cells, leading to uncontrolled inflammatory responses. Chen et al. [9] provided novel evidence from an epigenetic perspective, finding that the Foxp3 gene locus—a key transcription factor for Treg cells—exhibits higher methylation levels in elderly asthmatic patients. This may represent an intrinsic molecular mechanism leading to Treg cell functional exhaustion, offering a fresh perspective on how age-related immunosenescence specifically impacts asthma pathogenesis.

The dysregulation of T-cell immunity in elderly asthma does not occur in isolation but is deeply integrated with a systemic background of “immunosenescence.” Hallmarks of immunosenescence include a constricted naïve T-cell repertoire, expansion of memory T cells, and the accumulation of terminally differentiated senescent T cells due to replicative senescence. These senescent T cells secrete a spectrum of pro-inflammatory cytokines and chemokines, contributing to a state of chronic low-grade inflammation (“inflammaging”), which is also significant in the initiation and progression of asthma. A prospective study by Jones et al. [10] further revealed that the clonal expansion of specific T cells in the elderly, driven by persistent viral infections (e.g., cytomegalovirus), occupies immunological space and weakens the capacity to respond to new antigens. Concurrently, this expansion can exacerbate airway inflammation through bystander effects, potentially representing an additional important mechanism underlying the increased susceptibility and severity of elderly asthma.

In summary, the pathogenesis of elderly asthma is closely associated with functional disturbances in Th cell subsets, manifested by persistent Th2 responses, enhanced Th17 activity, and declining Treg cell function—all superimposed on the broader context of immunosenescence. A deeper understanding of this complex immune network will provide a solid theoretical foundation for developing precise immunomodulatory therapeutic strategies tailored to this unique patient population.

### Research progress on the role of Th cells in elderly bronchial asthma

#### Th1 and Th2 cells

The pathogenesis of elderly asthma involves a complex immune imbalance, particularly the interplay between Th1 and Th2 cells. In an aged rat model (22–24 months old), Zhao et al. [11] demonstrated that PM2.5 exposure activates both Th1 and Th2 immune response pathways concurrently. Significantly elevated levels of the Th2-related cytokines IL-4, IL-5, and IL-13, as well as the Th1-related cytokines interferon gamma (IFN-γ), IL-2, and tumor necrosis factor alpha (TNF-α), were observed in elderly asthmatic rats, indicating a mixed Th1/Th2 immune response. This mixed inflammatory pattern positively correlates with airway hyperresponsiveness, eosinophilic and neutrophilic infiltration, and exacerbation frequency, suggesting that simultaneous activation of Th1 and Th2 pathways is associated with increased disease severity in elderly asthma.

As noted in a systematic review by Mandlik et al. [12], Th2 cells orchestrate eosinophilic airway inflammation by secreting IL-4, IL-5, and IL-13, with IL-4 promoting IgE class switching, IL-5 sustaining eosinophil survival, and IL-13 regulating mucus hypersecretion, goblet cell metaplasia, and airway remodeling. Conversely, while IFN-γ produced by Th1 cells can antagonize Th2 responses, in severe asthma characterized by low type 2 inflammation, a predominance of IFN-γ-positive Th1 cells is paradoxically associated with glucocorticoid resistance. Through bioinformatics analysis, Liu et al. [13] identified 32 senescence-associated differentially expressed genes in neutrophil-dominant asthma (NA). These genes were primarily enriched in pathways related to cytokine-mediated signaling, lipopolysaccharide response, and regulation of T cell activation. Elevated expression of genes such as CCL20 and NLRP3 closely correlates with glucocorticoid resistance. CCL20 (CC chemokine ligand 20) serves as a potent chemoattractant for Th17 cells and dendritic cells via C-C motif chemokine receptor 6 (CCR6); its upregulation in neutrophil-dominant asthma enhances the recruitment of IL-17-producing cells, thereby amplifying neutrophilic inflammation and contributing to steroid insensitivity. NLRP3 (NOD-, LRR-, and pyrin domain-containing protein 3), a core component of the inflammasome complex, activates caspase-1-dependent cleavage of pro-IL-1β and pro-IL-18, releasing mature IL-1β and IL-18 that sustain airway inflammation and promote fibrosis. Elevated NLRP3 expression in elderly asthmatic airways thus serves as both a biomarker of inflammasome activation and a potential therapeutic target; NLRP3 inhibitors (e.g., MCC950, dapansutrile) are being investigated for chronic inflammatory diseases and could be repurposed for steroid-resistant elderly asthma.

**Figure 1.**
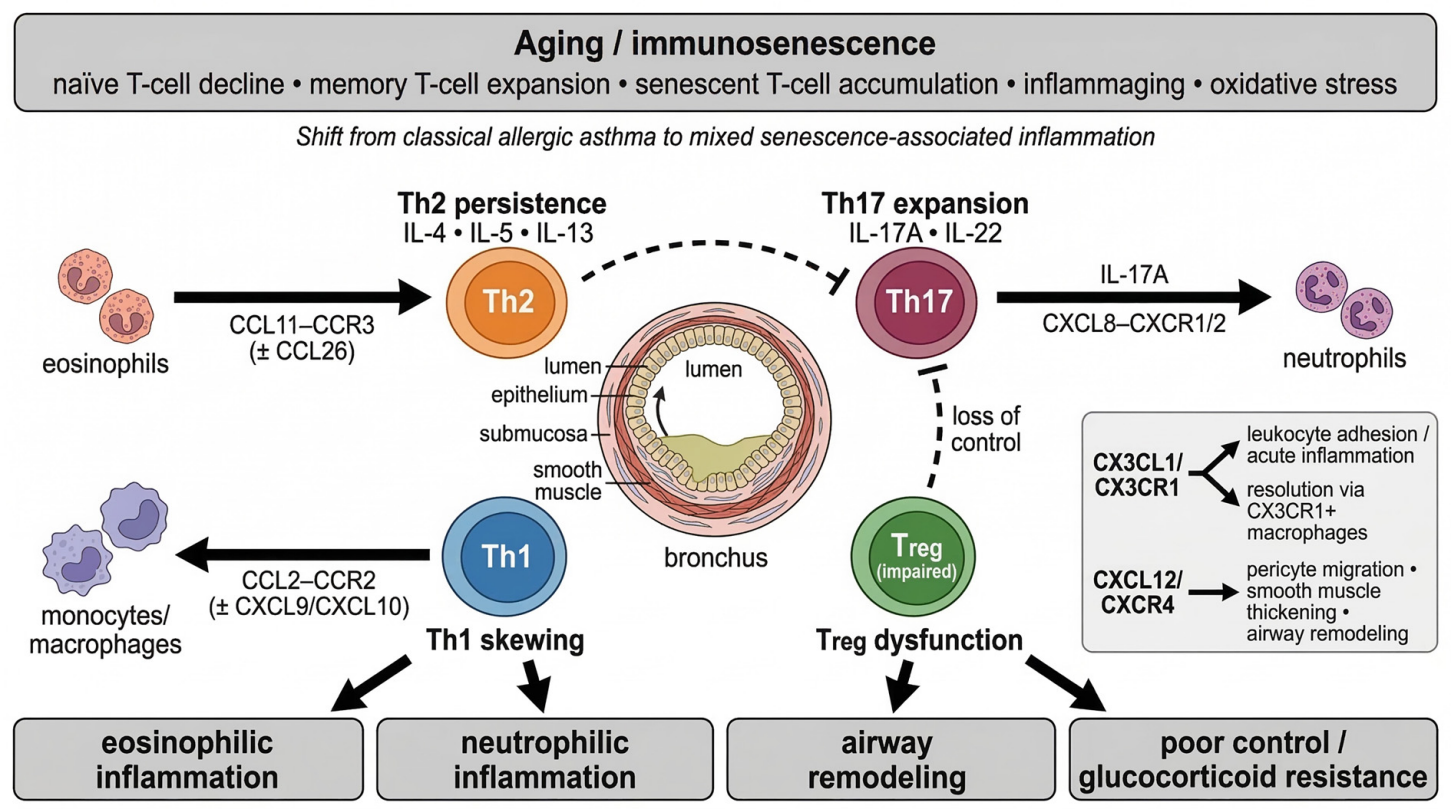
**Immunosenescence-driven Th-cell and chemokine network in elderly asthma.** Aging-associated immune remodeling shifts elderly asthma from a predominantly allergic paradigm toward a mixed inflammatory state characterized by persistent Th2 signaling, enhanced Th17/Th1 activity, and impaired Treg suppression. Major chemokine axes recruit eosinophils, neutrophils, monocytes/macrophages, and remodeling-associated cells, thereby promoting airway inflammation, tissue remodeling, poor disease control, and glucocorticoid resistance. Abbreviations: Th: helper T cell; Treg: regulatory T cell.

In eosinophilic asthma (EA), 12 senescence-associated genes (SAGs) were identified, primarily involving T cell-mediated immune regulation and viral response. Among these, C-C motif chemokine ligand 26 (CCL26; eotaxin-3) and C-X-C motif chemokine ligand 14 (CXCL14) are particularly relevant. CCL26 is a selective eosinophil chemoattractant signaling exclusively through C-C motif chemokine receptor 3 (CCR3); its overexpression in eosinophilic asthma directly drives eosinophil recruitment into the airways and correlates with disease severity. CXCL14, identified as an epithelial-derived chemokine, promotes eosinophil accumulation by binding to C-X-C motif chemokine receptor 4 (CXCR4) on eosinophils; it is upregulated by allergen exposure and may contribute to the persistent eosinophilic inflammation observed in some elderly patients. Both CCL26 and CXCL14 are detectable in sputum and serum, making them candidate biomarkers for identifying type-2-high elderly asthma that could benefit from anti-IL-5/IL-5Rα or anti-CCR3 biologics. Moreover, neutralizing antibodies targeting CXCL14 have shown efficacy in preclinical models, suggesting a novel therapeutic avenue for eosinophilic asthma in the elderly. These findings indicate that different inflammatory phenotypes of elderly asthma exhibit distinct patterns of Th cell dysregulation. In summary, the pathogenesis of elderly asthma is characterized by a disturbed Th1/Th2 equilibrium. Alterations in the expression of SAGs may significantly contribute to the suboptimal response of elderly patients to conventional therapies. Targeted interventions against specific Th cell subsets and their related senescence markers hold promise as novel strategies for treating elderly asthma. A conceptual framework integrating these immune shifts and chemokine networks is illustrated in Figure 1.

#### Th17 cells

Building upon the overview in Figure 1, recent research has confirmed that the immune-inflammatory pathway mediated by T helper 17 (Th17) cells and their signature cytokine, IL-17, is closely associated with airway neutrophilic infiltration, mucus hypersecretion, and airway remodeling in elderly asthma. Multiple clinical studies have elucidated the central role of the Th17 response. Building on the Th17/Treg imbalance introduced in Section 2.1, we focus on the specific effector mechanisms of IL-17A that drive airway pathology. The Th17/IL-17A axis has been shown to correlate with neutrophilic infiltration, mucus hypersecretion, and airway remodeling in elderly asthma. Using flow cytometry and enzyme-linked immunosorbent assay (ELISA), Xie et al. [14] found that the proportion of Th17 cells in peripheral blood and serum levels of IL-17A were significantly elevated in elderly asthmatic patients compared to healthy controls. This elevation correlated negatively with both predicted forced expiratory volume in one second (FEV1%) and the Asthma Control Test (ACT) score, indicating that higher Th17 activity is associated with poorer lung function and worse disease control. A foundational study by Wang et al. [15] further elucidated the underlying cellular mechanism, demonstrating that in human airway epithelial cells (from non-elderly donors) stimulated with dust mite extract, IL-17A upregulated the expression of CXCL8 (IL-8) by activating the nuclear factor kappa B (NF-κB) signaling pathway, thereby potently recruiting neutrophils to the airways. This provides direct experimental evidence explaining the neutrophilic inflammation commonly observed in elderly asthma. Although this study did not utilize elderly-specific models, the mechanism is highly conserved and likely contributes to the neutrophilic inflammation observed in elderly asthma.

Airway remodeling is a key pathological change leading to the progressive decline in lung function among elderly asthmatic patients. In a murine asthma model, Zhang et al. [16] confirmed that IL-17A directly promotes the transformation of airway fibroblasts into highly secretory myofibroblasts and accelerates the deposition of extracellular matrix proteins (such as collagen I and fibronectin). This study elucidated a novel, inflammation-cell-independent pathway through which IL-17A directly drives airway fibrosis, highlighting its important role in the irreversible airway damage characteristic of elderly asthma.

As detailed in Section 2.1, immunosenescence creates a permissive background for Th17/Treg imbalance. A clinical study [17] comparing asthmatic patients across age groups confirmed that the elderly asthma group exhibits a significantly increased Th17/Treg ratio in peripheral blood, directly correlating with persistent, difficult-to-control inflammation.

Given the pivotal role of the Th17 pathway, targeting it has emerged as a potential therapeutic strategy. In their review, Kim et al. [18] systematically summarized a series of preclinical studies and early clinical trials, noting that monoclonal antibodies directly targeting IL-17A (e.g., secukinumab) show promise for specific neutrophilic phenotypes of severe asthma. However, challenges related to heterogeneous efficacy remain. The development of more precise biomarkers is needed to identify the subset of elderly asthmatic patients most likely to benefit from IL-17-targeted therapies.

## The role of chemokines in the pathogenesis of elderly asthma

### Relationship between chemokines and the pathogenesis of elderly asthma

C-C motif chemokine ligand 11 (CCL11; eotaxin-1) primarily mediates the recruitment of eosinophils through its receptor CCR3. A clinical study by Ulambayar et al. [19] involving 142 patients with asthma, including 68 aged ≥65 years, found that serum levels of CCL11 were significantly higher in elderly asthmatic patients with persistent eosinophilia compared to both healthy controls and elderly asthmatic patients without eosinophilia. Furthermore, CCL11 levels positively correlated with peripheral blood eosinophil counts and the severity of airway obstruction. This indicates that the CCL11/CCR3 axis remains a key pathway driving eosinophilic inflammation and influencing lung function in the elderly population.

Neutrophilic airway inflammation is a prominent feature of elderly asthma. Chavez et al. [20] reported significantly elevated levels of CXCL8 (IL-8) in the bronchoalveolar lavage fluid of elderly asthmatic patients. Further *in vitro* experiments demonstrated that airway epithelial cells derived from these patients, when subjected to senescence-associated stimuli, persistently hypersecreted CXCL8 via the p38 mitogen-activated protein kinase (MAPK) signaling pathway, which strongly recruited neutrophils through the CXCR1/2 receptors. Their study was the first to directly link airway senescence, CXCL8 hypersecretion, and neutrophilic inflammation, providing a novel explanation for the pathogenesis of elderly asthma.

Monocyte-derived macrophages are critical contributors to chronic airway inflammation. A recent clinical study (2023) by an international research team [21] demonstrated that the concentration of C-C motif chemokine ligand 2 (CCL2; monocyte chemoattractant protein-1, MCP-1) in the sputum of elderly asthmatic patients was significantly higher than in healthy elderly individuals and positively correlated with the proportion of CD14+CD16+ intermediate monocytes in the sputum. These activated monocytes migrate into the airways, differentiate into macrophages, and release various inflammatory factors that amplify and sustain local inflammation, thereby contributing to the chronic nature of asthma in the elderly.

Airway remodeling is a major factor leading to irreversible lung function impairment in elderly asthmatic patients. Lavandoski et al. [22] identified CCL11 as a key chemokine associated with asthma, essential for eosinophil migration to the lungs in asthmatic patients. They noted its high expression in the airway epithelial cells of atopic asthmatics and found that its levels correlated with shortened leukocyte telomere length in asthmatic children. In a systematic review, Duchesne et al. [23] assessed the therapeutic potential of targeting chemokine receptors such as CCR3, CCR4, and C-X-C motif chemokine receptor 2 (CXCR2) in asthma. They concluded that while therapies targeting individual chemokine pathways have shown limited efficacy in the diverse asthma population, CXCR2 antagonists may be promising in biomarker-selected phenotypes, particularly in neutrophilic elderly asthma. To provide a concise overview of the major chemokine axes discussed, Table 2 summarizes key chemokines, their receptors, cellular targets, specific roles in elderly asthma, and corresponding therapeutic strategies.

**Table 2:** Key chemokine-receptor axes in elderly asthma: Mechanisms and therapeutic implications

**Chemokine**	**Primary receptor(s)**	**Main cellular sources**	**Principal target cell(s)**	**Receptor expression on target cells** **(verified)**	**Role in elderly asthma pathogenesis**	**Therapeutic implications**
CCL11 (Eotaxin-1)	CCR3	Airway epithelial cells, fibroblasts, endothelial cells [22]	Eosinophils	Yes (confirmed by flow cytometry and immunohistochemistry) [22]	Drives persistent eosinophilic inflammation; correlates with airway obstruction and exacerbation frequency	CCR3 antagonists (preclinical); may benefit elderly patients with persistent eosinophilia
CXCL8 (IL-8)	CXCR1, CXCR2	Airway epithelial cells, macrophages, neutrophils [20]	Neutrophils	Yes (CXCR1/2 expression confirmed on human neutrophils) [20]	Mediates neutrophilic inflammation; linked to steroid resistance and airway senescence	CXCR2 antagonists (e.g., danirixin, AZD5069); candidate for neutrophilic elderly asthma
CCL2 (MCP-1)	CCR2	Airway epithelial cells, smooth muscle cells, monocytes [21]	Monocytes, macrophages	Yes (CCR2 expression confirmed on CD14+ monocytes) [21]	Recruits inflammatory monocytes; amplifies and sustains chronic airway inflammation	CCR2 antagonists (e.g., PF-04136309); under investigation for chronic inflammatory diseases
CX3CL1 (Fractalkine)	CX3CR1	Airway epithelial cells, endothelial cells, smooth muscle cells [26, 27]	Macrophages, T cells, smooth muscle cells	Yes (CX3CR1 expression confirmed on macrophages and T cells) [26, 27]	Dual role: promotes leukocyte adhesion during acute inflammation; facilitates resolution via CX3CR1^+^ macrophages; chronic dysregulation contributes to remodeling	CX3CR1 modulators (preclinical); context-dependent targeting required
CXCL12 (SDF-1)	CXCR4, CXCR7	Fibroblasts, endothelial cells, bone marrow stromal cells [29, 30]	Pericytes, fibrocytes, stem cells	Yes (CXCR4 expression confirmed on pericytes and fibrocytes) [30]	Drives pericyte migration → airway smooth muscle thickening; also involved in tissue repair and stem cell homing	CXCR4 antagonists (e.g., AMD3100/plerixafor); stem cell-based therapies targeting SDF-1/CXCR4 axis

Note: The column titled “Receptor Expression on Target Cells (Verified?)” indicates whether the target cells listed in the adjacent column have been shown to express the corresponding chemokine receptor in published studies (citations provided). Methods for verifying expression include flow cytometry, immunohistochemistry, and/or transcriptomic analysis, as reported in the cited literature. Abbreviations: CCL11: C-C motif chemokine ligand 11; CCR3: C-C chemokine receptor 3; CXCL8: C-X-C motif chemokine ligand 8; CXCR1: C-X-C chemokine receptor 1; CXCR2: C-X-C chemokine receptor 2; CCL2: C-C motif chemokine ligand 2; MCP-1: Monocyte chemoattractant protein 1; CCR2: C-C chemokine receptor 2; CX3CL1: C-X3-C motif chemokine ligand 1; CX3CR1: C-X3-C chemokine receptor 1; CXCL12: C-X-C motif chemokine ligand 12; SDF-1: Stromal cell-derived factor 1; CXCR4: C-X-C chemokine receptor 4; CXCR7: C-X-C chemokine receptor 7.

### Research progress on the role of chemokines in elderly bronchial asthma

####  CC chemokines

CC chemokines serve as crucial regulators of immune cell migration and play an increasingly recognized role in the chronic airway inflammation and immunosenescence associated with elderly asthma. Unlike classic Th2-type inflammation, elderly asthma often displays a complex pattern of inflammatory cell infiltration, predominantly orchestrated by various CC chemokines. CCL5 (RANTES) is a broad-spectrum chemokine capable of attracting T cells, eosinophils, and monocytes. Mandlik et al. [12] described a mechanism in which airway dendritic cells present allergen peptides to naïve T-cell receptors via MHC class II molecules. The co-stimulatory interaction between dendritic cells and T cells promotes T-cell differentiation toward a Th2 phenotype. In this context, C-C motif chemokine ligand 17 (CCL17) and C-C motif chemokine ligand 22 (CCL22) act as Th2-cell-attracting chemokines, while CCL11, C-C motif chemokine ligand 24 (CCL24), and CCL26 serve as eosinophil-selective chemoattractants, collectively driving a type 2-high inflammatory phenotype. Chaudhary et al. [24] noted that the CC chemokine family plays a pivotal role in the pathogenesis of elderly asthma. As small chemoattractant cytokines interacting with G protein-coupled receptors, chemokines exert a central influence on aging and age-related diseases, primarily affecting multiple organ systems through inflammatory responses (inflammaging), macrophage recruitment, and coordinated trafficking of immune cells. Significant functional differences in SAGs are observed across various inflammatory phenotypes of elderly asthma. Wang et al. [25] found that eosinophil-attracting chemokines such as C-C motif chemokine ligand 1 (CCL1) and CCL26 are highly expressed in eosinophilic asthma, recruiting eosinophils via the CCR3 receptor and exacerbating airway inflammation. Mechanisms including age-related alterations in immune function, increased oxidative stress, persistently activated inflammatory responses, and telomere shortening collectively promote aberrant expression of CC chemokines, aggravating the pathological progression of elderly asthma.

#### C-X3-C motif chemokine ligand 1 (CX3CL1)

CX3CL1 (fractalkine), the sole member of the CX3C chemokine family, has unique dual biological functions: its membrane-bound form mediates leukocyte adhesion, while its soluble form exerts chemotactic activity. Its only known receptor, C-X3-C motif chemokine receptor 1 (CX3CR1), is expressed on various immune cells and plays a complex yet crucial role in modulating airway inflammation in elderly asthma. Airway epithelial cells are a significant source of CX3CL1 in the lungs [26]. Endothelial cells, airway smooth muscle cells, and fibroblasts also produce CX3CL1 under inflammatory conditions [28]. Key stimuli inducing CX3CL1 expression include pro-inflammatory cytokines such as TNF-α and IFN-γ [26], as well as allergen exposure [27]. In the context of aging, increased oxidative stress and chronic low-grade inflammation (“inflammaging”) may further upregulate CX3CL1 production, contributing to the persistent inflammation observed in elderly asthma [24].

The expression of CX3CR1 and the recruitment of inflammatory cells exhibit distinct patterns across different phases of airway inflammation. During the acute inflammation phase, CX3CR1 is expressed on Th1 cells, cytotoxic T cells, and inflammatory monocytes [26]. These CX3CR1+ cells are actively recruited to the airways, where membrane-bound CX3CL1 mediates their adhesion to endothelial and epithelial surfaces, facilitating their infiltration into airway tissues. This process amplifies the inflammatory response and recruits effector cells that drive airway hyperresponsiveness and tissue damage.

Conversely, during the recovery/resolution phase, a distinct population of CX3CR1+ macrophages plays a critical immunoprotective role. Utilizing single-cell RNA sequencing and mass cytometry, Moon et al. [27] observed dynamic reprogramming of alveolar macrophages after allergen inhalation, characterized by a significant increase in a CX3CR1+ macrophage subset expressing high levels of C1q family genes. CCL26, secreted by airway epithelial cells, activates this macrophage subset via binding to CX3CR1, inducing C1q secretion that promotes eosinophil clearance. Both CX3CR1 deficiency and airway epithelial cell-specific knockout of CCL26 resulted in delayed resolution of eosinophilic pulmonary inflammation, confirming the CCL26-CX3CR1 pathway as a critical mechanism for inflammation resolution. Thus, the same receptor (CX3CR1) can mediate opposing functions depending on the cellular context and phase of inflammation, promoting effector cell recruitment during the acute phase while facilitating pro-resolving macrophage activity during recovery.

A study by Godwin et al. [26] demonstrated that CX3CR1 deficiency leads to severe lung function impairment, accompanied by uncontrolled increases in neutrophils, eosinophils, and inflammatory monocytes, indicating that CX3CL1/CX3CR1 balances inflammatory cell infiltration and preserves lung function. Moon et al. [27] further elucidated the cellular mechanisms underlying this protective role. Their use of single-cell RNA sequencing and mass cytometry revealed dramatic reprogramming of alveolar macrophages following allergen inhalation, characterized by a significant increase in a CX3CR1+ macrophage subset expressing elevated levels of C1q family genes. Epithelial-derived CCL26 activates this macrophage subset via CX3CR1 binding, inducing C1q secretion that promotes eosinophil clearance. Both CX3CR1 deficiency and airway epithelial cell-specific CCL26 knockout resulted in delayed resolution of eosinophilic pulmonary inflammation, confirming the CCL26-CX3CR1 axis as a critical pathway for inflammation resolution. Thus, the CX3CL1/CX3CR1 axis is not intrinsically pro-fibrotic; rather, its sustained dysregulation in the aging lung—where resolution mechanisms are impaired—may shift the balance toward chronic inflammation and airway remodeling. A systematic review [28] further revealed that CX3CL1 is expressed in airway smooth muscle cells and is involved in regulating airway remodeling and inflammatory cell recruitment. Glucocorticoids can modulate CX3CL1 expression levels in airway tissues, offering new insights into the molecular mechanisms of steroid therapy in asthma.

In summary, the age-related tendency toward immune imbalance and diminished inflammation resolution capacity in elderly asthmatic patients underscores the potential of the CX3CL1/CX3CR1 axis as a therapeutic target, given its dual role in maintaining inflammatory balance and promoting resolution. Future mechanistic studies specifically focused on the elderly population are warranted to explore the clinical applicability of selective CX3CR1 modulators in improving airway inflammation and promoting disease remission in elderly asthma.

#### Stromal cell-derived factor-1 (SDF-1)

Stromal cell-derived factor-1 (SDF-1), also known as C-X-C motif chemokine ligand 12 (CXCL12), is a pivotal member of the CXC chemokine family. It plays essential roles in immune cell chemotaxis, tissue repair, and inflammation regulation by binding to its sole known receptor, CXCR4 [29]. Recent studies suggest that the SDF-1/CXCR4 axis significantly influences airway inflammation and remodeling in elderly patients with bronchial asthma.

The SDF-1/CXCR4 signaling axis has been identified as a crucial molecular pathway driving pathological structural changes associated with airway remodeling. Bignold et al. [30] demonstrated in a mouse model of chronic allergic airway inflammation (using young adult mice) that lung pericytes significantly upregulated CXCR4 expression in response to chronic inflammatory stimuli and exhibited enhanced chemotaxis towards its ligand, CXCL12. Although this study did not involve aged animals, the pericyte-CXCR4 axis represents a conserved remodeling pathway that is likely exacerbated in the aging lung. This increased migratory capacity was correlated with the detachment of pericytes from lung microvessels and their accumulation around the airway wall, resulting in thickening of the airway smooth muscle layer and heightened respiratory distress. Local treatment with the CXCL12-neutralizing ligand LIT-927 effectively inhibited pericyte detachment and migration, presenting a novel therapeutic strategy for targeting airway remodeling.

Systematic reviews further illuminate the multifaceted functions of SDF-1 in respiratory diseases. SDF-1 serves not only as a potent chemoattractant that recruits immune cells to sites of inflammation but also plays a role in regulating tissue repair and maintaining immune homeostasis. Research conducted by Biyani et al. [31] revealed that SDF-1 directs mesenchymal stem cell migration, regulates osteogenesis and chondrogenesis, promotes angiogenesis, and modulates the inflammatory microenvironment. In immune responses, SDF-1 aids in regulating leukocyte trafficking and the spatial positioning of immune cells within tissues, which is critical for maintaining the dynamic balance of airway inflammation in asthmatic patients.

Investigations into bone marrow-derived mesenchymal stem cell (BMMSC) therapy provide new insights into the role of SDF-1 in asthma. BMMSCs express various cytokine receptors, including those for SDF-1 and CXCR4, enabling them to respond to inflammatory signals and migrate to sites of airway inflammation. Research by Yuan et al. [32] demonstrated that BMMSCs can be recruited to injury sites through the homing properties mediated by the SDF-1/CXCR4 axis, exerting anti-inflammatory effects. Additionally, exosomes derived from BMMSCs can modulate immune responses, promote macrophage M2 polarization, inhibit Th2 cytokine expression, and alleviate airway inflammation and remodeling via the Wnt/β-catenin pathway.

In elderly asthmatic patients, age-related alterations in immune function and chronic inflammatory states underscore the pronounced regulatory role of the SDF-1/CXCR4 axis. Therapeutic strategies targeting this signaling pathway, such as CXCR4 antagonists or stem cell-based regenerative medicine approaches, may offer new treatment options for improving airway remodeling and controlling inflammation in elderly patients. Future clinical studies specifically targeting the elderly population are essential to evaluate the long-term efficacy of SDF-1-targeted therapies in enhancing lung function and quality of life.

#### CXCR4 receptor

C-X-C chemokine receptor type 4 (CXCR4) is a significant G protein-coupled receptor that primarily binds to its ligand CXCL12/SDF-1, playing vital roles in immune cell homing, inflammation regulation, and tissue repair. Recent research indicates that CXCR4 has a multidimensional regulatory role in eosinophil recruitment, airway inflammation, and remodeling processes in elderly bronchial asthma [33, 34].

The CXCR4-mediated signaling pathway has been recognized as a crucial mechanism for inflammatory cell infiltration in the airways. A recent study [35] found that airway epithelial cell-derived CXCL14 promotes eosinophil migration to the airways by binding to CXCR4 on the eosinophil surface. Stimulation with Alternaria alternata, a major environmental allergen, induced airway epithelial cells to secrete CXCL14 through reactive oxygen species produced by the mitochondrial oxidative phosphorylation complex (particularly complex II). Concurrently, allergen stimulation upregulated CXCR4 expression on eosinophils. This interplay between epithelial CXCL14 secretion and eosinophil CXCR4 upregulation is significant for eosinophil infiltration during allergic airway inflammation.

While CCR3 serves as the primary receptor for eosinophil chemotaxis, CXCR4 functions as a complementary receptor involved in cell recruitment to inflammatory sites. Research by Rao et al. [36] demonstrated that eosinophils express various chemokine receptors, including C-C motif chemokine receptor 1 (CCR1), C-C motif chemokine receptor 4 (CCR4), and CXCR4. Although the direct role of CXCR4 in eosinophil migration is limited, it can exert auxiliary regulatory functions within specific inflammatory microenvironments. Furthermore, eosinophils exhibit considerable heterogeneity in allergic airway inflammation, encompassing resident and inflammatory subsets. These subsets display distinct chemokine receptor repertoires: resident eosinophils preferentially express CCR3 and respond robustly to eotaxins (CCL11, CCL24, CCL26), while inflammatory eosinophils upregulate CXCR4 and CCR4, making them responsive to CXCL12 and Th2-associated chemokines CCL17/CCL22, respectively [36]. This differential receptor expression enables selective recruitment of resident eosinophils during homeostasis and early allergic responses, whereas inflammatory eosinophils are preferentially recruited during sustained or severe inflammation.

In systematic analyses of pathophysiological mechanisms in lung diseases, the axis formed by CXCR4 and its ligand CXCL12 has been confirmed to play significant roles in various respiratory disorders. As noted by Komolafe et al. [37], CXCR4 not only regulates airway inflammation but also participates in the airway remodeling process in asthma. The CXC chemokine family can be categorized into ELR+ and ELR- groups based on the ELR motif, which effectively attract neutrophils and lymphocytes/monocytes, respectively. CXCR4, as a crucial CXC receptor, interacts with CXCL12 to modulate airway inflammatory cell recruitment, fibrosis progression, and angiogenesis, rendering it a potential therapeutic target.

For elderly asthmatic patients, age-related immunosenescence and chronic low-grade inflammatory states may influence the expression and function of CXCR4. Targeting the CXCR4 signaling pathway with therapeutic strategies such as CXCR4 antagonists or anti-CXCL14 antibodies may offer new treatment options to enhance airway inflammation control and reduce eosinophil infiltration in elderly patients. Future studies specifically focused on the elderly population are necessary to elucidate the precise mechanisms of CXCR4 within the context of age-related immunological changes.

## Conclusion and future perspectives

This review has clarified the central roles of chemokine-receptor axes in elderly asthma, revealing that, against a backdrop of immunosenescence, non-Th2 pathways exemplified by the Th17/IL-17 and CXCL12/CXCR4 axes drive neutrophilic inflammation, airway remodeling, and glucocorticoid resistance. These mechanistic insights highlight several promising therapeutic strategies, including CXCR2 antagonists (e.g., danirixin) for neutrophilic asthma, IL-17A blockers (e.g., secukinumab) for steroid-resistant Th17-high phenotypes, CXCR4 modulators (e.g., AMD3100) to limit pericyte-mediated remodeling, and epigenetic Foxp3 demethylation to restore Treg function. However, a significant unmet need is the near-complete exclusion of adults aged over 75 years from biologic trials; dedicated studies focusing on this specific age group are urgently required to establish efficacy and safety in this distinct population. Concurrently, clinically applicable biomarkers—such as sputum CXCL8 for neutrophilic asthma, serum CCL11 for persistent eosinophilic inflammation, and Foxp3 methylation status for Treg dysfunction—offer a pathway to phenotype-guided therapy. Ultimately, a precision medicine framework tailored to the aging immune system, integrating clinical phenotype, inflammatory biomarkers, and senescence-associated molecular signatures, holds the greatest promise for improving outcomes in elderly asthma.

## Data Availability

All data generated or analyzed during this study are included in this published article. Further inquiries can be directed to the corresponding author.
